# Parents' Perspectives on XR Distraction During Pediatric Needle Procedures

**DOI:** 10.1002/pne2.70047

**Published:** 2026-09-16

**Authors:** Henrik Hjelmgren, Johanna Granhagen Jungner, Cecilia Bartholdson, Klas Kalholm, Anna Stålberg

**Affiliations:** ^1^ Astrid Lindgren's Children's Hospital Karolinska University Hospital Stockholm Sweden; ^2^ Paediatric Healthcare Science, Childhood Cancer Research Unit, Department of Women's and Children's Health Karolinska Institute Stockholm Sweden

**Keywords:** family‐centered care, needle‐related procedures, parental perspective, XR distraction

## Abstract

Parents play a crucial role in supporting their children during pediatric hospital visits, where needle‐related procedures are commonly performed. Both children and parents may experience distress related to needles, which can make these procedures more challenging. Adequate distraction, such as extended reality, may help to mitigate these potential difficulties. The aim of this study was to explore parents' perspectives on their child's use of extended reality as a distraction during a needle‐related procedure. In this exploratory and prospective mixed‐methods study, a total of 30 parents completed a study‐specific survey and 26 were interviewed. Quantitative data were analyzed using descriptive statistics, and qualitative data were analyzed using reflexive thematic analysis informed by Braun and Clarke. Parents generally reported positive experiences with XR distraction, with high mean scores for recommending the XR device to other children undergoing a similar procedure (VR 5.00; AR 4.86, scale 1–5). Parent‐reported child pain was low in both groups (mean 0.93 for VR and 1.38 for AR, NRS 0–10). Some differences in parents' responses between virtual and augmented reality were observed; however, the study was not designed or powered to detect differences between the two approaches. Nevertheless, parents perceived both approaches as supporting pain and anxiety relief during the needle‐related procedure. Five themes were developed through the qualitative analysis: A rewarding addition for the child, Redirection of the child's attention, The importance of the user perspective, Diversified emotional reactions among parents, and Modified roles for nurses and parents. In conclusion, parents viewed extended reality distraction as a valuable tool for supporting children during needle‐related procedures. They described its potential to alleviate distress, redirect attention, and provide moments of calm and reassurance for both children and adults. While some concerns were raised, the overall parental perspective was that extended reality can serve as a meaningful resource in clinical care. The findings suggest that future implementation should adopt a flexible, family‐centered approach that balances the technological benefits with the preservation of therapeutic relationships.

## 1 Background

Parents play a crucial role in easing child distress in any stressful situation. Visits to healthcare settings may further increase a child's need for parental support. Parents' presence has been shown to help reduce children's anxiety during healthcare encounters [1, 2, 3]. However, parents do not always have enough knowledge of what happens in a healthcare visit [4]. Accordingly, they are themselves in need of information and preparation by healthcare professionals (HCPs) [5, 6] as well as being allowed to be close to their child [7]. If worried or feeling distressed, parents risk transmitting such feelings to their children [8].

Within pediatrics, needle‐related procedures (NRPs) are common and represent routine tasks for HCPs. However, for children, NRPs are described as the most difficult experiences when in hospital [9, 10, 11]. Also, parents find such procedures to be distressing events, both prior to and during the procedure [12, 13]. Research shows that 20%–30% of an adult population express needle phobia [14]. Moreover, when their children undergo NRPs, negative emotions and unpleasant memories can be provoked in parents [13]. Parental distress is further exacerbated by witnessing their child's suffering [15, 16, 17, 18] and may intensify if they are required to physically restrain their child during the procedure [19, 20]. Furthermore, parents may face an ethical dilemma when their child is distressed or reluctant to participate in an NRP, while the procedure is nevertheless considered necessary for the child's diagnosis or treatment. Parents may feel torn between protecting their child from immediate distress and supporting the completion of the procedure [21, 22].

During pediatric healthcare visits, registered nurses and nursing assistants, henceforth addressed as nurses, contribute significantly by providing the children comforting interventions. Such interventions are of importance also for the parents, for them to be able to create trust in the nursing staff [7, 23]. Nurses have also previously described that it is a challenge to work with the family's during NRPs [24]. The adoption of family‐centered care, an approach widely implemented in pediatric healthcare, involves collaboration between HCPs and the family, the child, and the parents, respectively, with a focus on communication and support [25]. Parental involvement and shared responsibility for the child's care [26] enable parents to actively prepare and support their child throughout treatment and procedures [27]. One way to provide children comfort and by that, at least partly, alleviate parental vulnerability in the situation is the use of distraction. Distraction, whether active or passive, a non‐pharmacological cognitive strategy [28, 29, 30] helps children redirect their attention away from potentially frightening or painful procedures towards more enjoyable activities. Extended reality (XR), encompassing virtual reality (VR) and augmented reality (AR), is a relatively novel method of distraction. Despite its novelty, both VR and AR have shown promising results in reducing distress associated with painful procedures in pediatric care [31, 32, 33, 34]. VR provides full immersion, while AR enables the user to view the physical environment alongside digital elements.

This study forms part of a larger project aiming to explore experiences of XR distraction during NRPs from the perspectives of children, parents, and registered nurses. Previous publications from the project have described children's experiences of XR distraction, highlighting both positive experiences and individual variation [35], as well as registered nurses' experiences of opportunities and challenges related to XR use, including changes in nurse–child communication [36]. However, parents' perspectives have not yet been reported. The specific aim of this study was to explore parents' perspectives on their child's use of XR distraction during an NRP.

## 2 Methods

### 2.1 Design

In this study, an explorative and prospective mixed method approach was used. Quantitative data were gathered through self‐reported surveys. Semi‐structured interviews were conducted to collect qualitative data. The study is reported according to the COREQ checklist [37].

### 2.2 Setting and Recruitment

The study was conducted at a pediatric emergency department and across both outpatient and inpatient units at one of the largest tertiary pediatric hospitals in Sweden. This study formed part of a larger project involving a triad of stakeholders: Children, parents, and registered nurses. Parents were invited to participate in the study at the time of their child's inclusion and received verbal information about the study alongside their child. Parents became eligible for inclusion once their child had provided assent to participate. In this study, a parent was defined as either a biological or adoptive parent. Additionally, stepparents who had lived with the child for an extended period were also eligible to participate. In cases where both mother and father accompanied the child, both were permitted to take part in the study. Several children were accompanied by both parents, and in one case both parents participated. Another inclusion criterion was that parents had to be Swedish‐speaking, as interpreter‐assisted interviews were not feasible. Demographic information about the participating parents is presented in Table 1.

**TABLE 1:** Background data regarding participants and settings.

Categories	VR	AR	Total
Parents			
Father	3	7*	10
Mother	12	8*	20
Age			
Range	31–61	39–78	31–78
Median	40	45, 5	42
Setting			
Inpatient unit	5	5	10
Outpatient unit	5	5	10
Pediatric emergency department	5	4	9

* Two parents participated in the same AR observation; one survey was completed.

### 2.3 Data Collection

The NRPs of interest in this study were primarily peripheral intravenous catheter insertions, undertaken either for diagnostic purposes or because the child required intravenous treatment. All data were collected after the NRPs had taken place. Parents were invited to complete a study‐specific, self‐reported questionnaire developed by the research team in collaboration with a specialist in survey construction. The questionnaire explored parental experiences of their children's use of XR distraction. In this study, both VR and AR devices were employed as distraction tools. Children who experienced VR used the PICO 2 headset (PICO, China), with software developed by SyncVR (Netherlands) and Khora (Denmark), featuring a variety of mainly interactive games operated via a one‐handed controller. For AR, the Magic Leap 2 headset (Magic Leap, USA) was utilized, with software developed by DIMH‐byBrick (Sweden) and offering a customized AR game designed to be accessible for children of all ages. Children were given the option to begin using the XR device a few minutes before the procedure and continued using it throughout. However, use of the XR device was entirely voluntary and could be discontinued at any time if the child so wished. All interviews were conducted within the clinical setting, often in the same room where the needle procedure had just taken place. Two of the authors (H.H. and A.S.), along with one research assistant (IK), carried out all the interviews, which were audio‐recorded and subsequently transcribed using Microsoft Teams' AI transcription feature. The interviews lasted between 3.52 and 12.38 min.

The researchers involved in data collection and analysis were clinical nurse researchers in pediatric healthcare, with qualifications ranging from master's level to PhD and associate professor level. The team included researchers with experience as early adopters of XR technology in healthcare, as well as researchers with expertise in family‐centered care, children's rights, and qualitative research methodology. These professional backgrounds and experiences shaped the researchers' perspectives on the use of XR and parental involvement in pediatric care and were therefore considered throughout data collection, analysis, and reflexive discussions.

### 2.4 Data Analysis

The quantitative data from the self‐reported survey were transferred to a Microsoft Excel document on a server compliant with data protection regulations. A total of 30 parents participated in the study, while 29 surveys were completed. In one observation in the AR group, two accompanying parents participated, but only one survey was completed. Due to the small sample size, only descriptive statistics were used which are presented in Table 2 and outlined at the beginning of the Results section. For the qualitative data, reflexive thematic analysis, informed by Braun and Clarke [38, 39], was employed to identify patterns in shared meaning in the parents' perspectives on their children's use of XR distraction. Initially, the transcripts were divided into VR and AR groups. A preliminary analysis was conducted by two of the authors (K.K. and J.G.J.). A naive reading of the interviews was first undertaken to allow familiarization with the data. K.K. and J.G.J. subsequently coded a selection of interviews independently and discussed their coding and initial interpretations. These discussions were used to critically reflect on different interpretations of the data and to further develop the coding process, rather than to establish inter‐rater agreement. The remaining transcripts were then coded by K.K. and J.G.J. Following completion of the coding process, reflexive discussions were held among three of the authors (K.K., J.G.J., and H.H.). During these discussions, it became apparent that many of the codes initially developed for the VR and AR groups were similar. The analyses were therefore merged, and the developing patterns of meaning were reconsidered and reorganized. The additional authors (A.S. and C.B.) subsequently reviewed the merged analysis and thematic structure and proposed a reorganization and renaming of some of the themes. A further reflexive discussion involving all four authors resulted in further refinement of the themes. The final themes were developed through this iterative and reflexive process and were subsequently written up.

**TABLE 2:** Summary of the self‐reported survey.

Questions	VR (*n* 15 surveys)	AR (*n* 14 surveys)
Did you think that the XR‐device supported your child before the procedure?	Mean: 4.8	Mean: 3.43
Range 1–5 1 = not at all 5 = yes, definitely	1 = 0%	1 = 7%
	2 = 7%	2 = 21%
	3 = 0%	3 = 21%
	4 = 0%	4 = 21%
	5 = 93%	5 = 29%
How much pain did you feel your child experienced during the procedure itself?	Mean: 0.93	Mean: 1.38
NRS scale 0 = no pain 10 = worst pain ever	Min: 0	Min: 0
	Max: 7	Max: 5
		1 missing
Did you feel, when your child used the XR device, that it helped you feel less stressed during the procedure?	Mean: 3.93*	Mean: 2.69
Range 1–5: 1 = not at all 5 = yes, definitely	1 = 14%	1 = 29%
	2 = 0%	2 = 14%
	3 = 21%	3 = 36%
	4 = 7%	4 = 14%
	5 = 57% *1 parent chose not to answer but provided a written comment: Not relevant. I'm never stressed when it's time for needle‐related procedures.	5 = 7%
Would you like your child to have access to the XR device for the same or a similar procedure in the future?	Mean: 4.8	Mean: 4.07
Range 1–5: 1 = not at all 5 = yes, definitely	1 = 0%	1 = 8%
	2 = 0%	2 = 0%
	3 = 7%	3 = 21%
	4 = 7%	4 = 21%
	5 = 86%	5 = 50%
Would you recommend the XR device to other children undergoing the same or a similar procedure in the future?	Mean: 5	Mean: 4.86
Range 1–5: 1 = not at all 5 = yes, definitely	1 = 0%	1 = 0%
	2 = 0%	2 = 0%
	3 = 0%	3 = 0%
	4 = 0%	4 = 14%
	5 = 100%	5 = 86%

### 2.5 Ethical Considerations

This study received ethical approval from the Swedish Ethical Authority [dnr. 2023–03254‐01]. After having provided informed consent, the parents were given written information about the study and there was time for questions before study start. The parents were also informed of their right to withdraw at any time without facing any consequences for themselves or their children. Additionally, they were notified that the interviews would be audio‐recorded to facilitate transcription, which all gave permission to. To ensure confidentiality, the quotations used in the results section were labeled with general terms, such as relation to the child, setting, and the type of XR distraction used by the child.

## 3 Results

In this study, a total of 30 parents (mothers *n* = 20, fathers *n* = 10) participated in 29 instances where XR devices were used for distraction. Their participation was evenly distributed across the three settings, as was the children's use of the XR devices (VR *n* = 15; AR *n* = 14). Further background information is provided in Table 1.

Regarding the self‐reported survey, although there were overall positive responses, there were some differences in how the parents perceived their children's use of the XR devices. A vast majority (93%) of parents whose children used the VR device believed it was supportive prior to the start of the procedure. Parents in the AR group provided more varied responses, with 50% rating the device as supportive (scores 4 or 5), while 28% perceived the AR device as only slightly or not at all supportive (scores 1 or 2), compared to 7% in the VR group. Most parents in both groups considered the VR and AR devices to be useful tools for their children during the procedures (93% and 71%, respectively). Furthermore, they indicated that they would recommend the use of such devices for their own child, as well as for other children undergoing the same or similar procedures in the future. Regarding pain, parents perceived their children's pain levels as generally low in both groups, although some higher pain scores were reported (NRS scale 0–10, VR mean 0.93 and AR mean 1.38). When reflecting on their own experiences, parents in the VR group reported lower levels of stress during the procedures than parents in the AR group (see Table 2 for further details).

Of the 29 occasions on which XR devices were used, 26 interviews were conducted. Three interviews could not be performed due to clinical circumstances, for example, when parents needed to accompany their children to another location immediately after the NRPs were conducted. In addition, one interview recording could not be uploaded for transcription due to technical issues. Thus, 25 interview transcripts were available for analysis.

### 3.1 Themes

In the qualitative analysis, five themes were identified reflecting parents' perspectives on their children's use of XR distraction during NRPs: A rewarding addition for the child, Redirection of child attention, The user perspective is vital, Diversified emotional reactions among the parents, and Modified roles for nurses and parents (see Table 3).

**TABLE 3:** An overview of the reflexive thematic analysis.

Themes	Codes
A rewarding addition for the child	Valuable enhancements
	Distraction providers
	Enabling reduction of fear and pain
	Enjoyable supplements (for the child)
	Positive experiences (for the child)
Re‐direction of child attention	Diversion of child focus
	A complete capture of the child's awareness. The child feels pain but copes with the [needle] stick
	Beneficial not to watch the needle (VR)
	Allows an overview of the room – good/bad (AR)
The user perspective is vital	A need for tailored games
	XR not suitable for all children
Diversified emotional reactions among the parents	The child's XR use calms the parent
	The child's XR use does not calm the parent
	The child's XR use does not affect the parent
	XR suitable also for adult use
	XR use helps to calm the child
Modified roles for nurses and parents	Adjusted preparation and communication are required
	XR impacts the function of the parents

#### 3.1.1 A Rewarding Addition for the Child

In general, the XR devices were regarded by the parents as valuable enhancements to the procedure, and the parents were frequently amazed at how effectively their child coped with NRPs when supported by these novel tools.

I think this is a very good thing for children. I think it is very, very good. [Mother, outpatient unit, child using AR]

Although XR was sometimes viewed as a “luxury” addition, the parents appreciated the nurses' ongoing efforts to enhance the quality of care. They emphasized that the tools offered genuine distraction, helping their child feel safer and more at ease. Furthermore, parents perceived XR distraction as reducing expressions of pain and anxiety during procedures, as well as to mitigate negative associations with the hospital setting. Likewise, the use of XR devices was perceived to positively impact the child's response to the NRPs. This positivity seemed to stem from a mix of emotions. The child was seen as happier and more alert and procedures that had previously been distressing proceeded more smoothly. The parents perceived their child to find the virtual tools very enjoyable and helpful in reducing the intensity of the experience. Whether or not having previous experience of XR, the parents perceived that their child enjoyed exploring the virtual games. For children already interested in gaming, XR was especially fitting as it could help them downplay the seriousness of the situation.

She [the daughter] does not express any stress. She just lay there laughing. It was good. [Mother, PED, child using VR]

Laughter and enjoyment were interpreted as signs that the XR experience diminished the child's reactions to NRPs. These fun aspects of XR were seen as more engaging and effective and to provide more genuine distraction compared to more traditional distractions, such as smartphones or listening to music. However, some children were understood as being overly excited, which occasionally interfered with the procedure, for instance, if a child moved unexpectedly. Nonetheless, XR was considered a broadly positive experience. Even in cases where their child initially showed distress, the parents reported more rewarding outcomes after the XR devices were introduced.

According to her, it was very good, and she wanted to do it once more. Absolutely, if she needs another [needle] stick, she would like to use the device again. [Mother, inpatient unit, child using VR]

XR devices were often described as modern, “cool” tools well suited to today's young generation. They were believed to transform otherwise stressful experiences into playful ones, often creating the impression that time passed more quickly. Some parents noted that XR made it easier for nurses to locate veins, owing to the child being more relaxed.

#### 3.1.2 Redirection of Child Attention

The parents described that the XR devices enabled their child to disengage from the clinical environment. Moreover, the devices were understood to offer an engaging activity that enabled a shift of attention away from the real‐world situation towards the virtual games. By being immersed in the virtual environment, the child was perceived as less anxious and better able to forget about the procedure.

From my perspective, it seemed like she had other things to think about, she was focused on something else. When you focus on something else, you don't think about the actual [needle] stick. [Mother, PED, child using AR]

In some instances, parents emphasized that the XR games completely captured the children's awareness, regardless of whether VR or AR was being used. This deep level of engagement was likened to being in “another world”. While in this immersive state, the child often appeared not to want interruptions from the real world and showed no reaction at all to the NRPs. In cases where the child was understood as being partially distracted, but remained aware of the procedure, the responses were still perceived as less distressing, typically limited to a brief “ouch” or a facial grimace, but without crying or showing visible fear.

I noticed that you understood we were trying to distract you using the XR device. You are a clever boy. However, there was a [needle] stick, and afterwards everything was fine. [Father, PED, child using AR]

While parents generally agreed that both VR and AR were effective in redirecting children's attention, differences in their effects were observed. VR was found to completely block out visual cues from the hospital environment, which was considered particularly effective in reducing fear. Conversely, AR allowed the child to remain aware of the real‐world surroundings, which some parents believed was helpful for those who preferred to observe the procedure. However, for children with a fear of needles, this retained visibility was believed by the parents as counterproductive.

#### 3.1.3 The User Perspective Is Vital

Parents underlined the need for virtual games to be tailored in order to achieve the most effective distraction. Older children were described as finding the XR games more amusing, though the existing content of the AR game was often not age‐appropriate or sufficiently engaging. Furthermore, parents noted that a large variety of games was not necessary; rather, it was more important for a few games to align with the child's specific interests.

There is no need for a large number of games, just one, two, or three games related to the child's interest. For instance, if the child is very interested in dinosaurs. [Mother, PED, child using AR]

In some cases, XR was found to fail to deliver the intended effect, either because the child was perceived as struggling to be adequately immersed in the virtual environment or found it unengaging or even unsettling. Some parents observed that XR use could heighten, rather than alleviate, their child's anxiety. For children who did not exhibit fear related to needles, XR was perceived as a form of entertainment than a necessary distraction.

Although parents were generally positive about XR, some reported that their child being prone to suffer from nausea or dizziness was less likely to benefit from XR use. Concerns were also raised about the practicality of using the XR headsets for children who wore glasses, as the design could prevent them from fully participating. Moreover, because XR can significantly reduce a child's awareness of the procedure, some parents felt their child missed an opportunity to understand what was happening, something they, as parents, considered important. Additionally, some parents questioned the necessity of XR for children who had previously coped well without it. According to these parents, introducing XR in such situations might unintentionally signal that something alarming or distressing was about to occur, that is, something the healthcare staff were trying to conceal, thereby increasing anxiety in children who were not previously fearful.

One aspect to consider is that if your child doesn't find the procedure dramatic, perhaps just sees it as a [needle] stick, then the addition of such a device might make the situation feel “bigger”. There's something more about it. That's something to reflect on: will XR always be the right choice? [Mother, inpatient unit, child using AR]

#### 3.1.4 Diversified Emotional Reactions Among the Parents

Trypanophobia, that is, needle phobia, is not exclusive to children. The parents acknowledged that they found the NRPs easier and that they themselves felt calmer when their child used XR devices. Seeing their child happy and relaxed contributed to this sense of calm and reassurance.

I felt very calm. I could just focus on my own breathing. [Mother, PED, child using VR]

Although being calmer and reassured were the most frequently mentioned reactions, reductions in anxiety and stress were also expressed as an additional benefit of the child's use of XR was the redirection of the parents' attention. Although the primary focus in this study was on the parents' understanding of *their child's* experiences, some parents shared their own perspectives and suggested that XR could be beneficial also for adults as medical procedures can provoke anxiety in individuals of all ages, not just children. Accordingly, the parents emphasized a need also for them to redirect their attention from a potentially frightening event. Being distracted by the XR experience meant that parents did not need to observe the needle procedure closely, which they hoped would reduce the risk of transmitting their own needle phobia to their child. However, not all parents felt calm and reassured. For some, their child's use of XR did not lead to a sense of ease, and feelings of anxiety and worry persisted. Yet another group of parents felt largely unaffected by the introduction of XR. In particular, those with extensive experience of their child undergoing NRPs reported that XR had little to no impact on their own emotional responses. Some parents described XR simply as a fun addition, though it did not change how they felt during the procedure. These individuals felt that XR did not represent a significant improvement over the previously used method, e.g., standard of care.

Regarding their child, the parents generally perceived that the XR distraction was beneficial and that their child appeared calmer during the procedures. While the reduction in stress was sometimes described as modest, it was still noticeable. Compared to previous visits, the child was seen as more content, even before the procedure began.

Yes, she was calm and distracted. The AR use took the edge off an otherwise difficult situation. It was great. [Mother, outpatient unit, child using AR]

#### 3.1.5 Modified Roles for Nurses and Parents

When their child used XR devices, particularly VR, the parents described a need for adjusted preparation and nurse‐child communication. Likewise, they found that preparation and communication became more essential than otherwise. Nurses were described as needing to verbalize their actions differently when children were unable to see what was happening in the situation. Without adequate forewarning, parents identified a risk that their child might move unexpectedly, thereby jeopardizing the procedure. In contrast, prior explanation allowed procedures to proceed more smoothly.

The nurse started to draw the blood without informing her, and she twitched her arm. Later on, however, the nurse told her beforehand what would happen, and everything went well. Very well. [Mother, inpatient unit, child using VR]

Despite best efforts, the nurse‐child communication was sometimes perceived by the parents as being hindered when the XR devices were in use, as their child became deeply immersed in the games. The parents found these moments sub‐optimal and stressful.

XR also altered the roles of the parents. In previous NRPs, they sometimes had to physically restrain their child to complete a procedure. With XR, merely being present in the room was often sufficient. There was no longer a need for comfort or handholding. This shift reduced the pressure on parents and allowed them to observe their child coping more effectively, requiring less direct intervention. In addition, the child's use of XR was understood as creating a new opportunity for interaction, helping parents and children remain connected through shared moments of amusement and conversation.

Overall, the quantitative and qualitative findings were broadly consistent. The survey responses indicated generally positive parental experiences of XR distraction and low parent‐reported child pain, while the qualitative findings provided further insight into how parents perceived XR as supporting their children during the procedure. The qualitative data also expanded upon the survey findings by revealing variation in parents' own emotional responses and in their perceptions of VR and AR—aspects that were not fully captured by the quantitative measures.

## 4 Discussion

The aim of this study was to explore parents' perspectives on their child's use of XR distraction during an NRP. The findings revealed an overall positive parental view, focusing on aspects such as XR reducing children's procedural distress and offering enjoyable means of distraction. XR was also understood as reshaping the interaction between the three parties involved, placing different demands on adults, as nurses and parents, and altering their roles in the situation.

Although parents generally expressed positive views and strong reassurance regarding their child's use of XR, such reassurance may paradoxically convey underlying parental anxiety and fear to the child. This, in turn, could heighten rather than lessen the child's distress during NRPs [40]. Nonetheless, appropriate distraction methods promote coping behaviors and may reduce procedural fear and pain [16]. The findings of this study align with this: parents regarded XR as a valuable resource in the situation. While presenting the parental perspective on their child's use of XR distraction, the results support previous research showing a positive association between the use of XR and reduced levels of pain, fear, and anxiety in children undergoing NRPs [31, 32, 33, 34]. Additionally, parents understood XR as providing their children with opportunities to disengage from the clinical environment; whether by becoming immersed in the virtual world, forgetting about the real world, or by not associating hospital visits with negative experiences. The importance of considering the individual child's perspective is also supported by findings from the children's component of our larger project, where children described varied experiences of XR distraction and highlighted the importance of individual preferences [35]. This is consistent with parents' observations in the present study, who emphasized that the choice and content of XR should be adapted to the child's age, interests, and preferences.

Beyond being a clear resource for children during NRPs, XR distraction was also understood by parents as a resource for themselves, perceived in various ways. A common and important parental role during pediatric hospital visits is to provide comfort and support [1, 2, 27] and to advocate for the child, particularly for younger or non‐verbal children [14]. In this study, however, the need for parents to provide comfort or to defuse distressing situations was diminished. Instead, most parents found that their children were happily engaged with virtual games, largely unaware of what was happening in the real world. Parents consequently reported feelings of relief and reassurance, in part because of the reduced risk that the situation would escalate and require them to physically restrain their child, a scenario known to cause intense parental distress [19, 20]. It is well established that adults may experience pediatric medical traumatic stress [41]. NRPs can trigger negative emotions in parents by reawakening distressing past memories [12, 13, 15, 16, 17, 18]. Although not the primary focus of this study, some parents reflected on valuing XR distraction not only for their child's sake but also as a means of redirecting their own attention away from the procedure, thereby avoiding the transmission of their discomfort, or in some cases needle phobia, to their child. While overall parental perspectives on XR distraction were positive, a minority expressed indifference. These parents commonly reported that their children had already undergone numerous NRPs, reducing or eliminating the perceived need for distraction, for either parent or child, to cope with the procedure. Taken together, these findings indicate the importance of nurses remaining flexible in their encounters with children and parents. By listening carefully to the needs of both, and by fostering participation and collaboration, nurses can apply a family‐centered care approach that promotes individualized care [26, 42].

Although parents welcomed XR distraction overall, they raised concerns about the potential modification of nurse–child interactions. This shift was communicated both explicitly and implicitly as a potential risk to the quality of nurse–child communication. Parents therefore highlighted two important questions: (1) How can XR be implemented without diminishing interpersonal communication? and (2) Might XR interfere with the therapeutic nurse–child relationship? Similar reflections emerged in the nurses' study (part of the broader project), where nurses expressed concerns about being unable to provide their usual nursing care during these encounters. They also described a tendency for NRPs to shift from child‐centeredness to procedure‐centeredness [36]. According to Arthur et al. [43], novel technologies create opportunities for healthcare professionals but simultaneously pose new challenges. A recent paper emphasized the need for integration of technology grounded in responsibility, ethics, and equity, coupled with careful planning and real‐world testing prior to implementation in pediatric nursing [44]. Parents want to feel safe when entrusting their child to healthcare professionals. Combined with the challenges posed by novel technologies, this underscores the responsibility of healthcare providers to ensure that technology complements rather than replaces human interaction [45].

### 4.1 Strengths and Limitations

A strength of the qualitative component was the inclusion of parents from three different clinical settings, which contributed to a broader range of experiences and perspectives on the use of XR distraction. However, given the small sample size and the lack of power to detect differences between groups, the inclusion of different settings should not be interpreted as a strength of the quantitative component or as supporting generalisability of the quantitative findings. Another strength was the involvement of all authors at different stages of the analysis, with reflexive discussions contributing to the development and refinement of the analysis. Furthermore, as the study formed part of a larger project using the same methods, all authors were familiar with the data collection process. The inclusion of both mothers and fathers in the interviews also contributed to a more comprehensive understanding of parental experiences.

Limitations included the fact that some interviews were conducted by a research team member with limited research experience, albeit skilled in XR technologies. Most interviews took place immediately after the NRP, with the child often present in the room, which may have influenced parental responses. Additionally, XR distraction in this study was research‐driven, with a researcher present during the procedure. In routine clinical settings, parents might play a more active role in supporting nurses with XR use. While the quantitative data indicated that parents generally reported more positive experiences with VR than with AR, the groups should not be directly compared. The study was primarily designed to explore participants' experiences and was not powered to detect differences between the VR and AR groups. Therefore, these quantitative findings should be interpreted with caution and considered exploratory.

### 4.2 Clinical Implications

Overall, parents regarded XR distraction as an effective adjunct in pediatric care, not only for reducing children's procedural distress but also for alleviating their own emotional burden. At the same time, the findings emphasized the need for careful implementation that preserves meaningful communication between nurses, children, and parents. By tailoring XR interventions to children's preferences and incorporating parental perspectives, healthcare providers can integrate these technologies into practice in ways that enhance human interaction and family‐centered care.

## 5 Conclusion

This study showed that parents generally perceived XR distraction as valuable support during children's NRPs. Parents reported that XR reduced their children's pain, fear, and anxiety, while also easing their own stress and sense of responsibility. However, some concerns were raised, including age‐appropriateness and the potential risk of diminished nurse–child communication when children became deeply immersed in the virtual environment. Taken together, these findings suggest that XR technologies hold promise as a complement to existing strategies in pediatric care. Their use should remain flexible, tailored to each child and family, and implemented in ways that preserve meaningful human interaction. Future work should continue to explore how XR can be integrated into family‐centered care, ensuring that technological innovation strengthens therapeutic relationships central to pediatric healthcare, rather than replacing them.

## Author Contributions

Henrik Hjelmgren and Anna Stålberg led the conceptualization of the study, the data collection, and curation. Anna Stålberg, Klas Kalholm performed the data analysis with input from Johanna Granhagen Jungner and Henrik Hjelmgren. Henrik Hjelmgren and Anna Stålberg wrote the first draft of the manuscript with input from all co‐authors. Klas Kalholm, Johanna Granhagen Jungner, and Cecilia Bartholdson were involved in review and editing and approved the final version for publication. All authors have materially participated in the research and manuscript preparation.

## Funding

The authors have nothing to report.

## Ethics Statement

This study received ethical approval from the Swedish Ethical Authority [dnr. 2023–03254‐01]. After having provided informed consent, the parents were given written information about the study and there was time for questions before study start.

## Conflicts of Interest

The authors declare no conflicts of interest.

## Data Availability

The data that support the findings of this study are available on request from the corresponding author. The data are not publicly available due to privacy or ethical restrictions.
